# Dengue Hemorrhagic Fever Causing Postpartum Hemorrhage and Hemophagocytic Lymphohistiocytosis in a Young Woman: A Case Report

**DOI:** 10.7759/cureus.53841

**Published:** 2024-02-08

**Authors:** Pakkiyaretnam Mayurathan

**Affiliations:** 1 University Medical Unit, Teaching Hospital Batticaloa, Batticaloa, LKA; 2 Department of Clinical Sciences, Faculty of Health-Care Sciences, Eastern University of Sri Lanka, Batticaloa, LKA

**Keywords:** dengue hemorrhagic fever, postpartum hemorrhage, hemophagocytic lymphohistiocytosis, steroids, pregnancy

## Abstract

Dengue viral infection can present as a spectrum of disorders ranging from uncomplicated fever to dengue shock syndrome. Dengue fever during pregnancy or delivery is associated with serious complications during pregnancy, especially severe postpartum hemorrhage (PPH) following delivery. Dengue is an uncommon and highly fatal cause of secondary hemophagocytic lymphohistiocytosis (HLH). Both PPH and HLH in a pregnant woman lead to unfavorable outcomes even with appropriate treatment. Here, we report the case of a 28-year-old woman who presented with PPH and HLH following dengue hemorrhagic fever and completely recovered with appropriate treatment.

## Introduction

Dengue is an endemic viral infection in Sri Lanka. It can present in various ways, including uncomplicated dengue fever (DF), dengue hemorrhagic fever (DHF), and dengue shock syndrome. Dengue infection during pregnancy is associated with significant morbidity and mortality to the mother and the fetus [1]. Postpartum hemorrhage (PPH) with prolonged bleeding is the most serious complication among mothers with dengue infection [2].

Hemophagocytic lymphohistiocytosis (HLH) secondary to dengue infection is an uncommon manifestation. DHF in pregnancy complicated with HLH on top of PPH is highly fatal even with appropriate treatment [3,4].

HLH is a severe hyperinflammatory hyperferritinemic syndrome induced by activated macrophages, cytotoxic T cells, and natural killer (NK) cells. It is a rare clinical manifestation, and its causes can be classified into primary (hereditary/familial) or secondary (acquired). Primary HLH is usually seen among children and is caused by mutations in the genes that are responsible for the production of cytotoxic T cells and NK cells. The involved genes include *STX11*, *SH2D1A*, *PRF1*, *UNC13D*, and *ITK* [4]. Infections, rheumatological conditions, and malignancies are common secondary causes of HLH. Viral infections are most common among the infective causes, especially Epstein-Bar virus, human immunodeficiency virus, and cytomegalovirus. Bacteria, parasites, and fungi can also cause HLH [5]. Dengue viral infection is a well-recognized cause of HLH [3,4,6]. Juvenile arthritis, systemic lupus erythematosus, leukemias, lymphomas, and solid tumors are other common causes of secondary HLH [5-7].

Persistent fever, splenomegaly, pancytopenia, low fibrinogen level, high triglyceride level, and, most importantly, very high ferritin levels are the hallmarks of HLH in clinical practice [4,5]. Bone marrow biopsy is helpful to confirm HLH.

Rotational thromboelastometry (ROTEM) is a good guide in PPH to effectively manage patients without many complications. Specifically, it can guide clinicians in avoiding prolonged shock and shock-related ischemic events [8]. Recombinant activated factor VII (rFVIIa) is another novel therapeutic agent to manage most acute life-threatening bleeding conditions successfully, even in PPH [9].

## Case presentation

A 28-year-old pregnant woman with a period of amenorrhea of 38 weeks was admitted to the tertiary care hospital with a three-day history of fever. The fever was associated with arthralgia and myalgia and mild headache without chills or rigors. She denied cough, chest pain, difficulty breathing, postural giddiness, or any bleeding manifestations. Her urine output was normal. However, she had one episode of loose stool on the day of admission without any nausea, vomiting, or abdominal pain. She felt normal fetal movements throughout. It was her first pregnancy complicated with gestational diabetes mellitus. The patient was only on diet control with acceptable blood sugar control. There were no other significant medical problems, known allergies, or similar fever-related illnesses among the family members.

On admission (day three of fever), her weight was 68 kg. She was comfortable and febrile with a temperature of 38.8°C. There were no rashes or ankle edema. Her pulse rate was 118 beats/minute with good volume. Her capillary refilling time was less than two seconds. Her blood pressure was 110/60 mmHg and there was no postural hypotension. Abdominal examination was unremarkable except for pregnancy with a symphysis fundal height of 38 cm. All other respiratory, cardiovascular, and neurological examinations were normal.

Admission cardiotocography was normal. Paracetamol 1 g was administered for fever, and appropriate monitoring was arranged, including monitoring of pulse rate, blood pressure, pulse pressure, temperature, fluid balance chart, and fetal heart rate charts as per local and national guidelines. Immediate investigations revealed a hemoglobin (Hb) of 11.2 g/dL, packed cell volume (PCV) of 34.1%, white blood cell count (WBC) of 8,640/mm^3^, and platelet of 132,000/mm^3^ with positive dengue NS1 antigen. An urgent inward ultrasound scan showed no evidence of organomegaly or plasma leakage. Further, aspartate transaminase (AST) and alanine transaminase (ALT) were 49 U/L and 36 U/L, respectively, with normal C-reactive protein (CRP) and serum creatinine. DF was confirmed and a total fluid intake of 100 mL per hour with three-hour urine output measurements were planned. She was hemodynamically stable with normal urine output.

The next day (day four of the fever), she maintained all her hemodynamic parameters within the normal range including urine output without any fever spikes. Her repeat Hb was 12.8 g/dL with a PCV of 34.8%, WBC of 6,270/mm^3^, and platelet of 108,000/mm^3^. The repeat ultrasound did not show any evidence of plasma leakage. AST was 150 U/L and ALT was 72 U/L. The same management plan continued with 100 mL of fluid intake per hour.

On the following (day five of the fever) morning, she was hemodynamically stable with Hb of 11.6 g/dL with PCV of 34.2%, WBC of 4,700/mm^3^, and platelet of 55,000/mm^3^. However, ultrasound revealed evidence of plasma leakage as pericholic fluid collection with mild ascites. The diagnosis of DHF was made and an appropriate DHF monitoring chart was initiated at 07:00 while monitoring hourly urine output. At this time, 10 mL of 10% calcium gluconate was given intravenously over 10 minutes. Two hours later, the patient developed labor pain and was taken to the labor room as per the obstetrician’s recommendation. Blood grouping, cross-matching, urgent ROTEM, and a hematologist’s opinion were requested. A total of 100 mL of normal saline per hour was initiated with oxytocin (syntocinon) at the beginning of the labor. Urgent full blood count showed Hb of 10.5 g/dL with PCV of 31.4%, WBC of 9,130/mm^3^, and platelet of 40,000/mm^3^. In addition, her pulse rate increased to 138 beats/minute, and her blood pressure dropped to 90/60 mmHg. One unit of red cell concentrate and five units of platelets were transfused while monitoring the DHF chart and labor. With blood transfusion, her blood pressure increased to 110/70 mmHg and manual PCV increased to 33%.

At 13:26 she delivered a healthy female infant with a birth weight of 3.26 kg. The baby’s Apgar score was 10/10 at one minute, and vitamin K 1 mg was given to the neonate at birth. However, immediately after delivery, the mother started to experience heavy postpartum bleeding and her pulse rate went up to 184 beats/minute with blood pressure dropping to 64/48 mmHg. A Bakri postpartum balloon was inserted in the endometrial cavity by the obstetrician to control the bleeding. She needed a transfusion of 500 mL of 40% dextran, another three units of fresh blood, five units of platelets, and 150 mL of cryoprecipitate as per the hematologist’s opinion based on the ROTEM along with syntocinon within the next two hours to maintain the systolic blood pressure at more than 100 mmHg. Further, 1 g of tranexamic acid, 10 mg of vitamin K, 1.2 g of co-amoxiclav, and 500 mg of metronidazole intravenously were administered. In addition, 4 mg of rFVIIa was given (at 60 μg/kg body weight) intravenously as per the multidisciplinary team (MDT) decision. There was no urine output for two hours following delivery and she was transferred to the medical intensive care unit (MICU). After three hours of postpartum, she started to maintain normal blood pressure and acceptable urine output. Repeat full blood count showed Hb of 10.1 g/dL with PCV of 30.5%, WBC of 9,540/mm^3^, and platelet of 26,000/mm^3^. AST, ALT, and serum creatinine increased to 1,706 U/L, 297 U/L, and 1.3 mg/dL, respectively. Her activated partial thromboplastin time (APTT) and international normalized ratio (INR) were within normal limits. CRP increased to 54 mg/L. As she was hemodynamically stable and maintained normal urine output, 100 mL of normal saline per hour was given initially for six hours in the MICU. After eight hours of postpartum, the bleeding stopped and the fluid was switched to 60 mL of normal saline and 40 mL of oral fluid per hour. Estimated blood loss during PPH was about 3-3.5 L. She maintained normal capillary blood sugar throughout. The Bakri postpartum balloon was removed and another close examination and suturing of episiotomy were done by the obstetrician.

**Figure 1:**
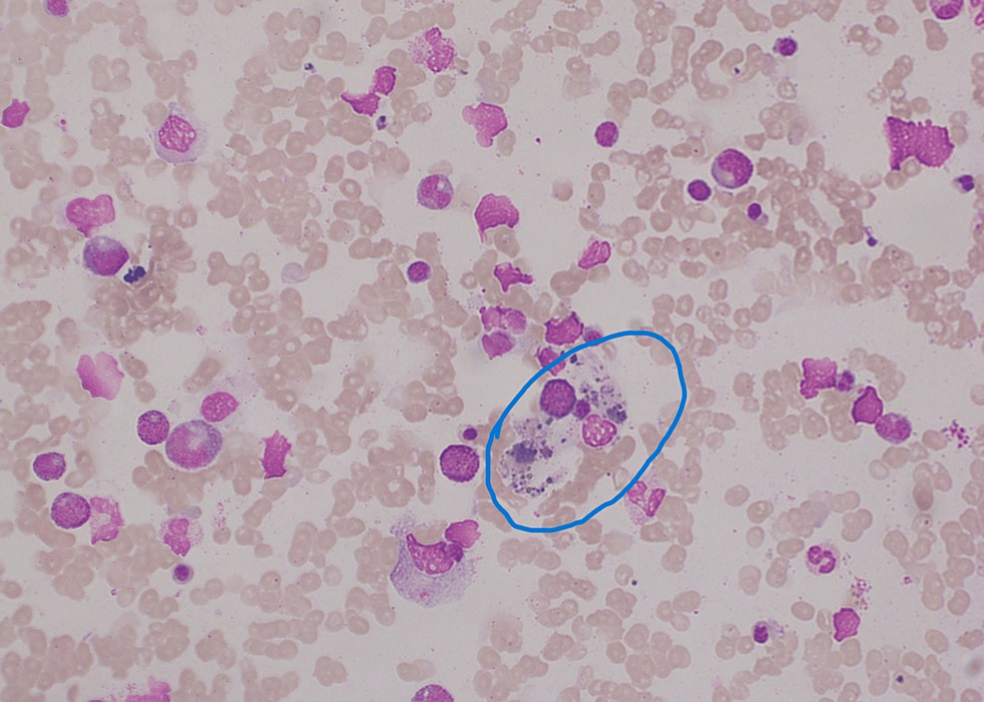
Bone marrow biopsy showing hemophagocytosis in our patient. Hemophagocytosis in the bone marrow biopsy is circled.

On the following day (day six of the fever), she was afebrile and maintained hemodynamic stability with normal urine output. She was able to breastfeed the baby as well. Her investigations revealed a drop in Hb of 8.5 g/dL with PCV of 25.5%, WBC of 9,250/mm^3^, and platelet of 23,000/mm^3^. AST was 1,179 U/L, ALT was 273 U/L, serum creatinine was 1.4 mg/dL, and CRP was 18 mg/dL. Repeat ROTEM after 24 hours was acceptable in terms of bleeding and clotting. However, ultrasound showed mild hepatosplenomegaly with a minimal amount of free fluid. At that point, we suspected HLH and the subsequent investigations confirmed high lactate dehydrogenase (LDH) of 759 IU/L (normal: 105-333 IU/L), high ferritin of 6,930 ng/mL (normal: 6.24-137.00 ng/mL), and high triglyceride level of 379 mg/dL (normal: <150 mg/dL). Her serum electrolytes, APTT, INR, reticulocyte count, and direct agglutination test were normal. Table 1 presents a summary of her investigations. Immediately the hematologist’s opinion was sought and an urgent bone marrow biopsy was performed. The bone marrow biopsy (Figure 1) showed evidence of hemophagocytosis and HLH was confirmed based on the 2004-HLH diagnostic criteria (Table 2). Immediately she was started on 1 g intravenous methylprednisolone. Total fluid intake was maintained between 80 and 100 mL per hour.

**Table 1:** Summary of investigations. *: Values mentioned within brackets in the first column represent the normal reference range of our hospital. Hb: hemoglobin; PCV: packed cell volume; WBC: white blood cells; CRP: C-reactive protein; AST: aspartate transaminase; ALT: alanine transaminase; APTT: activated partial thromboplastin time; INR: international normalized ratio; LDH: lactate dehydrogenase; TSH: thyroid-stimulating hormone

	On admission (day 3 of fever)	Day 4 of fever	Day 5 of fever	Day 6 of fever	Day 7 of fever	Day 8 of fever	Day 10 of fever	On discharge (day 14 of fever)	Review in 2 weeks after the discharge
Hb in g/dL (11.0–15.0)	11.2	12.8	11.6	8.5	8.9	9.3	9.7	11.0	11.4
PCV in % (37.0–47.0)	34.1	34.8	34.2	25.5	26.4	31.2	32.2	33.1	34.2
WBC in /mm^3^ (4,000–11000)	8,640	6,270	4,700	9,250	12,300	13,900	13,700	10,400	9,600
Platelets in /mm^3^ (150,000–450,000)	132,000	108,000	55,000	23,000	29,000	33,000	42,000	88,000	220,000
CRP in mg/L (<6)	6	10	54	18	20	16	15	8	6
AST in IU/L (15–37)	49	150	1706	1179	964	546	348	99	34
ALT in IU/L (12–78)	36	72	297	273	260	234	185	76	31
Serum albumin in g/L (34–54)	46	48	32	38	38	39	-	40	-
Total bilirubin in mg/dL (1.0–1.2)	-	-	-	1.0	1.0	-	-	1.0	-
Corrected calcium in mmol/L (2.2–2.6)	2.4	2.3	2.0	2.3	2.4	-	-	2.4	-
Serum sodium in mmol/L	143	140	137	140	139	141	144	143	139
Serum potassium in mmol/L	4.1	4.2	4.7	4.4	4.3	4.4	4.4	4.1	4.3
Serum creatinine in mg/dL (0.7–1.2)	0.8	0.8	1.3	1.4	1.1	1.0	0.9	0.9	0.8
APTT in seconds (30–40)	-	-	34	36	35	35	32	32	-
INR (0.8–1.2)	-	-	1.1	1.1	1.1	1.1	1.0	1.1	-
LDH in IU/L (105–333)	-	-	-	759	-	542	-	378	-
Reticulocyte count in % (<2)	-	-	-	1.2	-	-	-	-	-
Serum ferritin in ng/mL (6.24–137.00)	-	-	-	6,930	-	-	-	-	-
Serum triglyceride in mg/dL (<150)	-	-	-	379	-	-	-	-	-
Blood culture	-	-	Negative	-	-	-	-	-	-
Urine culture	-	-	Negative	-	-	-	-	-	-
TSH in mIU/L (0.4–4.0)	-	-	-	-	2.2	-	-	-	-
Dengue NS1 antigen	Positive	-	-	-	-	-	-	-	-
Dengue IgM antibody	-	-	-	-	-	Positive	-	-	-

**Table 2:** Hemophagocytic lymphohistiocytosis (HLH)-2004 diagnostic criteria.

The diagnosis of hemophagocytic lymphohistiocytosis (HLH) can be established if criterion 1 or 2 is fulfilled	
1	A molecular diagnosis consistent with HLH
2	Diagnostic criteria for HLH fulfilled with any five of the following eight features
	Fever ≥38°C
	Splenomegaly
	Cytopenias (affecting ≥2 of 3 lineages in the peripheral blood)
	Haemoglobin <9 g/dL
	Platelets <100,000/mm^3^
	Neutrophils <1,000/mm^3^
	Hypertriglyceridemia and/or hypofibrinogenemia
	Fasting triglycerides ≥265 mg/dL (≥3.0 mmo/L)
	Fibrinogen ≤1.5 g/L
	Hemophagocytosis in bone marrow or spleen or lymph nodes. No evidence of malignancy
	Low or no natural killer cell activity (according to local laboratory reference)
	Ferritin ≥500 μg/L
	sCD25 (soluble IL-2 receptor) ≥2,400 U/mL

From the next day (day seven of the fever) onward, she maintained hemodynamic stability including adequate urine output. Intravenous methylprednisolone was administered for three days along with antibiotics and supportive care and she was transferred to the medical ward for further management after three days of MICU care. Dengue IgM antibody was positive on day eight.

At the medical ward, oral prednisolone 1 mg/kg was initiated and all other supportive care was continued. Her Hb and platelets started to improve. She was discharged after 11 days of ward hospital management with oral prednisolone and omeprazole. Overall, a 1 mg/kg dose of oral prednisolone was given for two weeks, following which it was slowly tailed off over six weeks reducing the dose by 10 mg/week while monitoring her full blood count, fasting blood sugar, and CRP. We reviewed her weekly after discharge until postpartum six weeks.

She showed remarkable recovery at the completion of steroids. Her repeat ultrasound was normal at six weeks. We followed her monthly for up to three months and then discharged her from follow-up.

## Discussion

DHF during pregnancy can cause devastating outcomes, especially managing prolonged shock due to PPH during labor is the most challenging task for healthcare professionals. As we anticipated, our patient developed labor within the critical period of DHF. ROTEM is helpful in the management of PPH in DF. We managed her with blood, platelets, and cryoprecipitate based on the ROTEM findings. Repeated ROTEM is useful in the management if severe ongoing bleeding manifestations exist [8]. Furthermore, rFVIIa is highly effective in the management of PPH due to dengue infection [9]. Therefore, we decided to administer rFVIIa to our patient after an urgent MDT discussion. The cost of rFVIIa is high and it may not be available in most institutions in Sri Lanka. However, rFVIIa may save life.

Unfortunately, there is not enough literature evidence regarding the clinical manifestations and management of dengue-induced HLH during pregnancy. A high degree of suspicion is mandatory and lifesaving in terms of HLH. We suspected HLH because our patient’s blood counts continued to drop even with repeated blood transfusions and supportive care. Immediately, we performed an ultrasound scan and found hepatosplenomegaly, which is the cardinal feature of HLH. Other findings of high LDH, high ferritin, and high triglyceride levels supported our suspicion of HLH in our patient and we arranged an urgent bone marrow examination. Persistent fever, deterioration of cytopenia, hepatosplenomegaly, and increased serum ferritin level are the key clinical features of HLH. A high degree of suspicion is pivotal to investigate further to confirm HLH [4]. Our patient’s bone marrow biopsy also confirmed HLH (Figure 1).

HLH is diagnosed based on the 2004 diagnostic criteria [6]. At least five features out of eight are needed to confirm the diagnosis. Our patient met six features including fever, pancytopenia, splenomegaly, high ferritin, high triglycerides, and bone marrow evidence of hemophagocytosis, fulfilling the criteria to confirm the diagnosis. Management of HLH includes many medications including etoposide, cyclophosphamide, and steroids. Steroids are effective in secondary HLH and etoposide and other anticancer chemotherapies are useful in primary HLH. Intravenous dexamethasone and methylprednisolone are commonly used steroids in HLH due to infections in adults [6,10]. According to the literature, a short course of oral steroids following methylprednisone pulse therapy is effective with careful monitoring of clinical and biochemical parameters. Our patient showed remarkable improvement with steroids and other supportive care and completely recovered within six weeks.

## Conclusions

Diagnosing and managing PPH and HLH in a pregnant patient with DHF during pregnancy or delivery is highly challenging. Understanding the pathophysiological changes during pregnancy and postpartum is pivotal in early diagnosis. ROTEM investigation is usually helpful in managing PPH in pregnant women. Timely intervention with blood, blood products, and rFVIIa can help avoid fatal complications such as prolonged bleeding and shock in PPH.

Persistent fever, pancytopenia, and splenomegaly are important clinical features of HLH. A high index of suspicion and close monitoring of clinical parameters are crucial in managing HLH. An appropriate immunosuppressive agent, especially intravenous dexamethasone or methylprednisolone, is very effective in managing secondary HLH. Successful outcome depends on individualized patient care with an MDT approach and a dedicated management team.
